# ITRAQ-based quantitative proteomic analysis reveals that VPS35 promotes the expression of MCM2-7 genes in HeLa cells

**DOI:** 10.1038/s41598-022-13934-3

**Published:** 2022-06-11

**Authors:** Xian Hong, Tao Wang, Juan Du, Yu Hong, Cai-Ping Yang, Wei Xiao, Yang Li, Ming Wang, He Sun, Zhi-Hui Deng

**Affiliations:** 1 Laboratory of Protein Structure and Function, Institute of Medicine and Pharmacy, Qiqihar Medical University, Qiqihar, 161006 Heilongjiang China; 2 Department of Histology and Embryology, Basic Medical School, Qiqihar Medical University, Qiqihar, 161006 Heilongjiang China

**Keywords:** Biochemistry, Cancer, Cell biology, Molecular biology

## Abstract

Vacuolar protein sorting 35 (VPS35) is a major component of the retromer complex that regulates endosomal trafficking in eukaryotic cells. Recent studies have shown that VPS35 promotes tumor cell proliferation and affects the nuclear accumulation of its interacting partner. In this study, isobaric tags for relative and absolute quantitation (iTRAQ)-based mass spectrometry were used to measure the changes in nuclear protein abundance in VPS35-depleted HeLa cells. A total of 47 differentially expressed proteins were identified, including 27 downregulated and 20 upregulated proteins. Gene ontology (GO) analysis showed that the downregulated proteins included several minichromosome maintenance (MCM) proteins described as cell proliferation markers, and these proteins were present in the MCM2-7 complex, which is essential for DNA replication. Moreover, we validated that loss of VPS35 reduced the mRNA and protein expression of MCM2-7 genes. Notably, re-expression of VPS35 in VPS35 knockout HeLa cells rescued the expression of these genes. Functionally, we showed that VPS35 contributes to cell proliferation and maintenance of genomic stability of HeLa cells. Therefore, these findings reveal that VPS35 is involved in the regulation of MCM2-7 gene expression and establish a link between VPS35 and cell proliferation.

## Introduction

Retromer is an evolutionarily conserved complex that regulates endosomal protein trafficking in all eukaryotic cells^1^. In human cultured cells, retromer is required for the recycling of more than 100 integral plasma membrane proteins from the endosome to the cell surface^2^, and mediates the retrieval of multiple transmembrane proteins from the endosome to the trans-Golgi network (TGN)^3^. These retromer cargos include proteins involved in lysosomal degradation^4^, Wnt-dependent development^5^, autophagy^6^, neuronal morphogenesis^7,8^, nutrition^9^, glucose and metal ion transport^2^; thus, retromer is crucial for cellular homeostasis. The core of the retromer complex consists of the vacuolar protein sorting (VPS) proteins VPS35, VPS29 and VPS26; VPS35 acts as the central hub of this complex^10,11^. Dysfunctional VPS35 or retromer has been linked with neurodegenerative diseases such as Parkinson’s and Alzheimer’s disease^12^. Additionally, a recent study showed that VPS35 functions as a novel oncogene in liver hepatocellular carcinoma to promote the proliferation of hepatoma cells through the PI3K/Akt signaling pathway^13^. However, the detailed mechanisms underlying the function of VPS35 in cell proliferation and carcinogenesis are incompletely understood.

Retromer functions together with a large number of accessory proteins to regulate endosomal trafficking and retrograde vesicular transport^14^. The pentameric WASH (Wiskott-Aldrich Syndrome and SCAR Homolog) complex, which promotes endosomal branched actin polymerization via the actin-related 2/3 (Arp2/3) complex, is a key regulator of the retromer-dependent trafficking pathway. WASH tightly associates with 4 interacting proteins, including FAM21, SWIP, Strumpellin and CCDC53^15,16^. Retromer directly associates with the WASH complex through a direct interaction between VPS35 and the multiple leucine-phenylalanine (LFa motifs) repeats of FAM21^17^, and this interaction has been reported to be necessary for endosomal recruitment of the WASH complex^18–20^. Interestingly, our previous study indicated that VPS35 and several components of the WASH complex, including WASH and FAM21, are present in the nucleus^21^. Of note, VPS35 is required for the nuclear accumulation of FAM21^21^. However, whether VPS35 affects the abundance of other proteins in the nucleus remains to be elucidated.

Isobaric tags for relative and absolute quantitation (iTRAQ) is an advanced high-throughput quantitative proteomics technique with unique advantages, such as accurate quantification, a high identification rate and high-efficiency sample separation^22^. It has been used not only to study a range of organisms from bacteria to humans^23^, but also to examine proteins from different cell fractions, including cytoplasmic, membrane, nuclear, chromatin-bound, mitochondrial and secretome proteins^24–32^. In this study, we extracted nuclear proteins from VPS35 knockdown and control HeLa cell lines, and compared the nuclear proteomes of these cell lines using iTRAQ coupled with nanoscale liquid chromatography-tandem mass spectrometry (NanoLC–MS/MS). This iTRAQ approach led to the identification of several components of the minichromosome maintenance protein 2–7 (MCM2-7) complex, which are downregulated in VPS35-depleted cells. Moreover, these iTRAQ results were further validated by western blot and quantitative real-time PCR (qRT–PCR) analyses. Our results reveal a new role for VPS35 in the regulation of MCM2-7 gene expression, extending our understanding of VPS35 from its previous retromer-dependent role in endosomal cargo trafficking.

## Results

### Extraction of nuclear proteins from VPS35 knockdown stable cells

**Figure 1:**
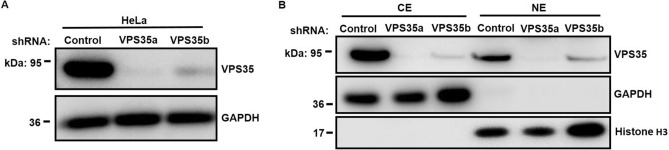
Validation of nuclear protein enrichment from HeLa stable cell lines. (**A**) Lysate prepared from cells stably transduced with specific lentiviral shRNAs (denoted a and b) or scrambled control was subjected to western blotting to examine the efficient knockdown of endogenous VPS35 in HeLa cells. (**B**) Cytosolic (CE) and nuclear extracts (NE) of HeLa cells stably expressing the indicated shRNA were analyzed by western blotting. GAPDH and histone H3 were used as cytoplasmic and nuclear markers, respectively.

To characterize the nuclear proteomic profile regulated by VPS35, we first generated stable VPS35 knockdown HeLa cell lines using lentiviral short hairpin RNA (shRNA) constructs as previously described^21^. As expected, the protein expression of VPS35 was markedly diminished in VPS35-silenced HeLa cell lines, as revealed by western blotting (Fig. 1A). The efficacy of the subcellular fractionation was determined by the results with GAPDH as a cytoplasmic marker and histone H3 as a nuclear marker. As shown in Fig. 1B, GAPDH was consistently present in cytoplasmic extracts (CE), while histone H3 was enriched only in the nuclear extracts (NE). Consistent with our previous observation^21^, VPS35 was observed in nuclear fraction.

### Protein identification

To increase reliability, two independent nuclear protein extracts from shControl and shVPS35a HeLa cells were performed as two biological replicates. In biological replicate 1 and 2, shControl and shVPS35a samples were labeled with 113 and 114 iTRAQ tags and with 116 and 119 iTRAQ tags, respectively. The final ratios of 114:113 and 119:116 indicated the relative abundance of the nuclear proteins in VPS35-depleted HeLa cells compared with the controls.

Based on the data acquisition, 529,358 spectra, 224,046 unique peptides and 3,186 proteins were identified (Supplementary Table S1). Based on the relative quantification and statistical analysis, a 1.2-fold change cutoff, iTRAQ ratios in two replicates > 1.2 for upregulation or < 0.83 for downregulation, was selected to denote a significant change. In total, 47 proteins were selected as potentially differentially expressed (Table 1). Of those, 27 proteins were downregulated and 20 proteins were upregulated in shVPS35a cells compared with the control cells (Table 1). A dramatic decrease in VPS35 was detected in both iTRAQ ratios, as expected (Table 1). Although our previous findings indicated that VPS35 depletion reduced FAM21 nuclear accumulation, FAM21 (B4DF48) was only found to undergo a mild decrease in the 119:116 iTRAQ ratio (Supplementary Table S1). This discrepancy might be due to technical differences in the detection of protein expression or limited MS sensitivity for low abundance proteins in the nucleus.

**Table 1:** Differentially expressed proteins in the nucleus identified by iTRAQ in VPS35-depleted HeLa cells compared to shControl cells.

Accession	Gene Name	Ratio 114:113	*P* value	Ratio 119:116	*P* value
**Proteins down-regulated in VPS35-depleted cells**					
Q9Y3B4	SF3B6	0.78	1.70E−03	0.81	1.02E−02
Q13885	TUBB2A	0.78	4.10E−04	0.74	2.40E−02
Q05639	EEF1A2	0.79	2.61E−04	0.67	2.12E−04
P14618	PKM	0.77	1.30E−61	0.62	5.73E−46
O75533	SF3B1	0.75	2.72E−03	0.73	1.05E−02
Q13867	BLMH	0.80	5.22E−06	0.73	1.57E−03
Q00610	CLTC	0.82	1.46E−03	0.83	2.40E−03
O75116	ROCK2	0.80	2.68E−03	0.82	1.49E−03
A0A087WVY5	CFAP57	0.79	4.00E−02	0.82	1.88E−02
P33316	DUT	0.77	2.22E−16	0.57	7.99E−17
O95155	UBE4B	0.75	8.45E−04	0.81	5.57E−03
A0A024R5Z9	PKM2	0.79	1.56E−05	0.74	1.54E−02
P04792	HSPB1	0.76	1.59E−19	0.66	5.23E−12
A1KZ92	PXDNL	0.81	2.14E−02	0.64	1.93E−02
F5H5D3	TUBA1C	0.80	2.24E−51	0.82	5.97E−17
Q96DG6	CMBL	0.72	2.72E−03	0.66	6.09E−03
Q9Y617	PSAT1	0.79	3.68E−06	0.66	5.88E−05
Q00796	SORD	0.80	3.92E−06	0.73	3.18E−10
A0A140VK07	N/A	0.82	2.78E−11	0.72	4.43E−11
A0A2C9F2M7	PHGDH	0.83	2.05E−22	0.62	5.48E−19
B2RBA6	MCM7	0.74	3.12E−04	0.68	4.57E−05
A8K521	MCM5	0.74	4.62E−05	0.80	3.32E−03
Q4ZG57	MCM6	0.76	1.34E−06	0.77	1.65E−04
B3KXZ4	MCM2	0.78	1.23E−18	0.78	2.05E−15
Q53FR4	VPS35	0.45	8.94E−19	0.72	9.48E−13
B4DFR2	N/A	0.80	5.88E−05	0.82	6.60E−03
A0A384NYT8	N/A	0.71	1.02E−24	0.74	5.07E−08
**Proteins up-regulated in VPS35-depleted cells**					
P84101	SERF2	1.22	6.71E−03	1.24	1.01E−02
Q99584	S100A13	1.27	3.46E−18	1.66	7.37E−42
Q96FQ6	S100A16	1.20	7.95E−10	2.43	1.15E−21
A0A087WUQ6	GPX1	1.42	4.19E−02	1.41	1.42E−03
Q8IV08	PLD3	1.67	1.28E−18	1.61	1.39E−12
P05114	HMGN1	1.23	3.93E−04	2.09	1.62E−10
Q9Y2Q3	GSTK1	1.31	2.28E−05	1.44	1.50E−08
Q13442	PDAP1	1.23	1.90E−05	1.31	3.62E−18
P06703	S100A6	1.24	2.67E−31	1.22	2.50E−18
Q00688	FKBP3	1.23	7.51E−12	1.28	4.34E−16
P26447	S100A4	1.30	1.92E−44	2.47	6.01E−79
A0A024RAI5	BIN1	1.44	2.54E−05	1.37	3.64E−04
P31949	S100A11	1.74	1.19E−11	1.75	9.70E−14
P21399	ACO1	1.25	5.34E−09	1.45	7.12E−23
Q15651	HMGN3 P	1.33	2.21E−03	1.68	3.22E−04
P05204	HMGN2	1.51	2.08E−12	1.77	1.02E−25
A0A024R9Q1	THBS1	1.29	7.52E−04	1.54	5.38E−03
A0A172Q3A8	FOLR1	1.21	1.72E−05	2.59	7.53E−14
H3BSM5	GABARAPL2	1.41	5.60E−04	1.68	4.29E−04
P25815	S100P	1.67	3.87E−03	1.91	6.89E−03

iTRAQ tags Assignment: Replicate 1: shControl-1, 113; shVPS35a-1, 114; Replicate 2: shControl-2, 116; shVPS35a-2, 119.

### GO analysis

**Figure 2:**
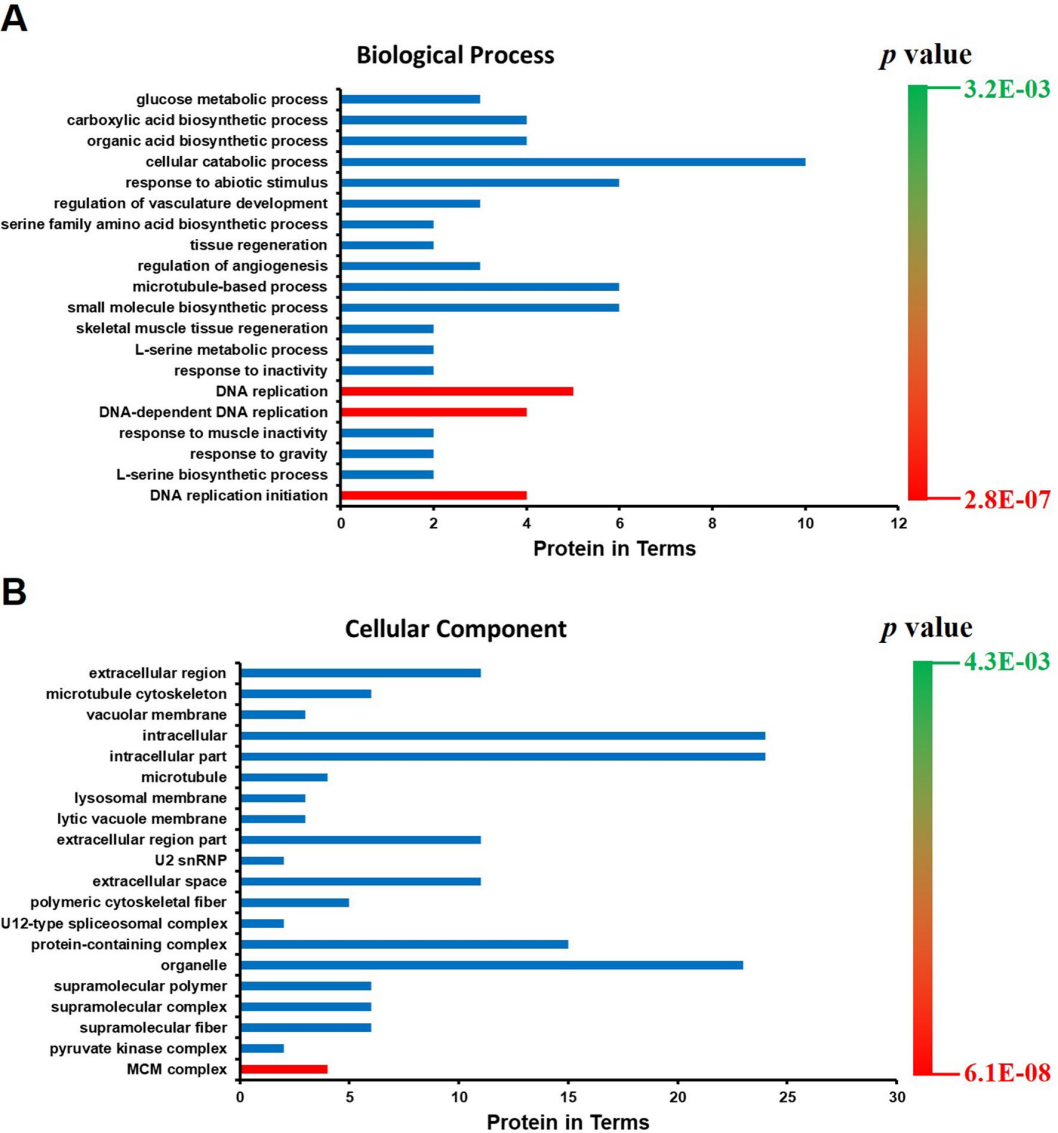
GO analysis of identified proteins downregulated in VPS35-depleted HeLa cells. GO analysis was performed according to biological process (**A**) and cellular component (**B**) by the PANTHER classification system. Red bars represent the DNA replication-related biological process (**A**) or cellular component (**B**).

To explore the potential function of VPS35, the PANTHER classification system was used to classify the differentially expressed proteins. Considering that VPS35 could affect nuclear protein abundance, we mainly focused on the downregulated proteins identified using the iTRAQ technique. GO analysis showed that the 27 downregulated proteins were mainly represented as 20 biological processes and 20 cellular components (Fig. 2A and B). Notably, GO analysis revealed an important biological process in the nucleus, DNA replication (Fig. 2A), and an MCM complex that is essential for DNA replication (Fig. 2B). The 4 members of MCM identified by iTRAQ included MCM2 (B3KXZ4), MCM5 (A8K521), MCM6 (Q4ZG57) and MCM7 (B2RBA6), which are present in the MCM2-7 complex. Therefore, iTRAQ and GO analysis results indicated that VPS35 might participate in regulating the expression of MCM2-7 proteins and in the DNA replication process.

### Validation of the protein expression of MCM2-7 by western blot analysis

**Figure 3:**
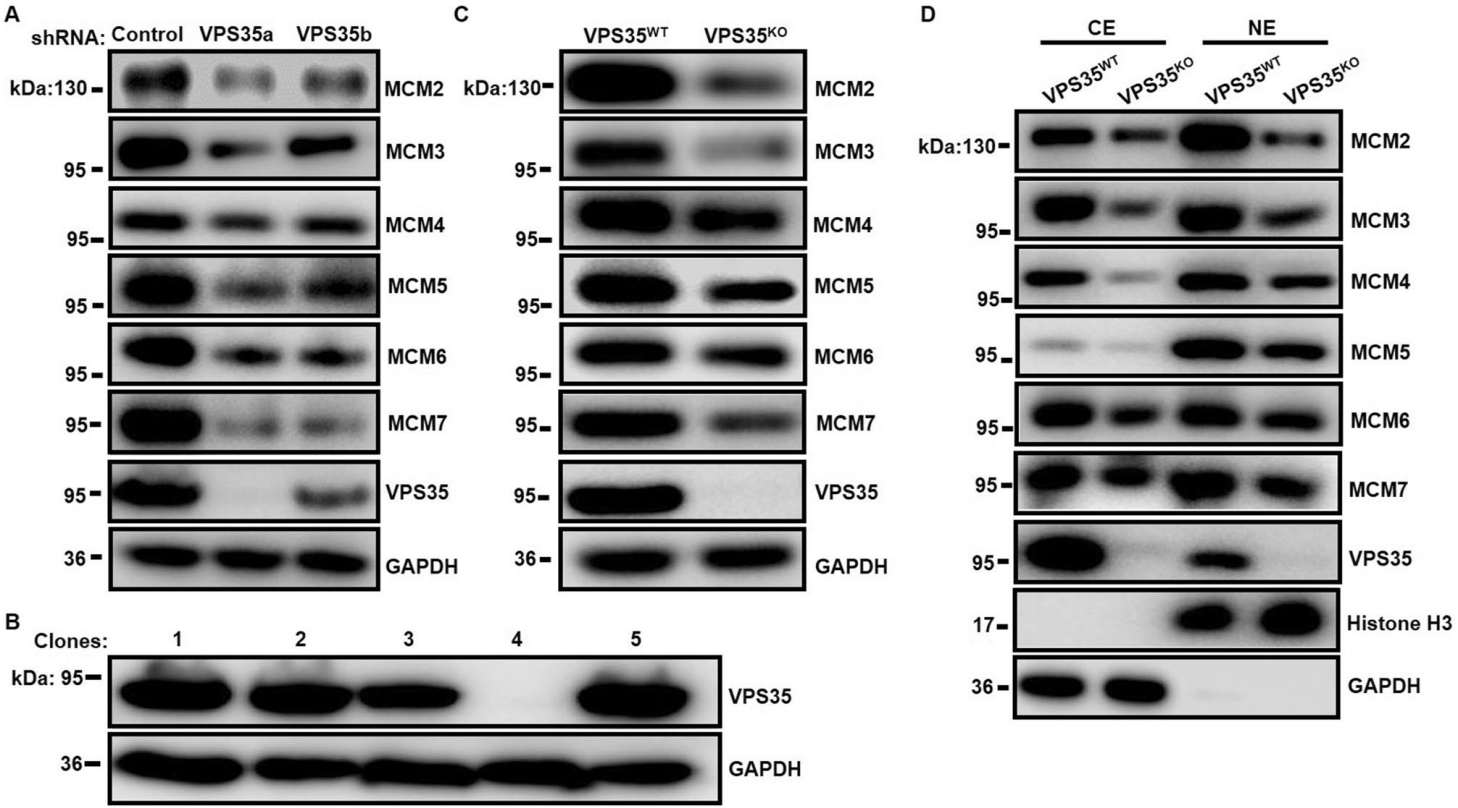
Determination of MCM2-7 protein expression by western blotting. (**A**) Expression of MCM proteins was detected in HeLa cells stably expressing the indicated shRNA. (**B**) VPS35 knockout cells were verified by western blotting, and representative blots are shown. (**C**) Expression of MCM proteins was detected in wild-type VPS35 (VPS35^WT^) and VPS35 knockout (VPS35^KO^) HeLa cells. (**D**) The cytosolic (CE) and nuclear extracts (NE) of VPS35^WT^ and VPS35^KO^ HeLa cells were analyzed.

Since the MCM2-7 complex is the core of the prereplication complex required for DNA synthesis during S-phase and is tightly associated with cell proliferation^33^, we focused on the validation of the expression of MCM2-7 proteins in HeLa cells lacking VPS35. Western blot analysis demonstrated that knocking down VPS35 markedly reduced the protein levels of all 6 members of the MCM2-7 complex (Fig. 3A). To further verify the protein expression, we constructed a VPS35 knockout (VPS35^KO^) HeLa cell line using the CRISPR/Cas9 system (Fig. 3B). Consistently, VPS35^KO^ cells displayed a similar decrease in MCM2-7 protein expression (Fig. 3C). Since VPS35 contributes to the nuclear localization of its interacting partner^21^, we next investigated whether VPS35 participated in the nuclear translocation of MCM proteins by preparing subcellular fractions. As shown in Fig. 3D, VPS35 knockout reduced the protein levels of MCM2-7 in the both cytosolic (CE) and nuclear fractions (NE), suggesting that VPS35 affected the expression of MCM2-7 proteins but not the nuclear translocation of these proteins.

### Validation of the mRNA expression of MCM2-7 by qRT–PCR

**Figure 4:**
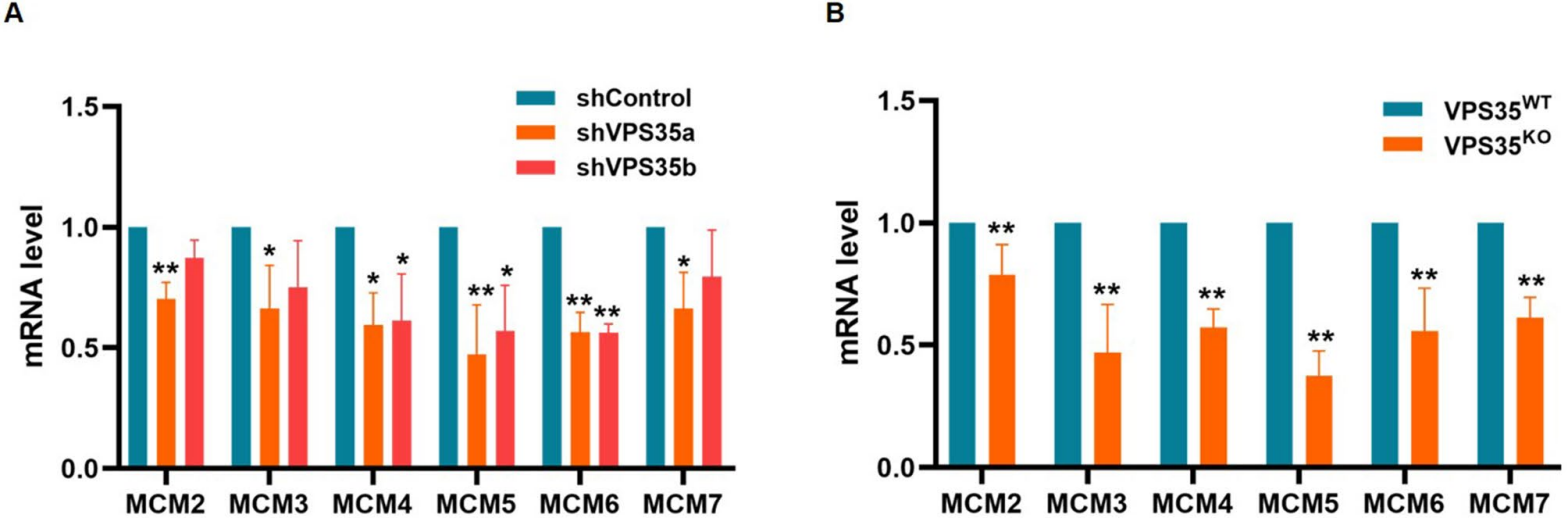
Evaluation of mRNA expression of MCM2-7 genes by qRT–PCR. (**A**) Basal mRNA expression of MCM genes was analyzed in HeLa cells stably expressing the indicated shRNA by qRT–PCR. The value of each gene expressed in shControl cells was normalized to 1, and that in shVPS35 cells was expressed as the mean ± S.D. derived from three independent experiments. (**B**) Basal mRNA expression of MCM genes was analyzed in VPS35^WT^ and VPS35^KO^ HeLa cells by qRT–PCR. The value of each gene expressed in VPS35^WT^ cells was normalized to 1, and that in VPS35^KO^ cells expressed was as the mean ± S.D. derived from three independent experiments. *, *p* < 0.05 vs. shControl or VPS35^WT^; **, *p* < 0.01 vs. shControl or VPS35^WT^.

Considering that the level of protein expression does not necessarily directly correlate with its mRNA level, we next performed qRT***–***PCR to test the mRNA expression. As shown in Fig. 4A, decreased mRNA levels of MCM4, MCM5 and MCM6 as well as VPS35 were observed in both VPS35 knockdown cell lines when compared with control cells. The mRNA expression of MCM2, MCM3 and MCM7 was significantly reduced in shVPS35a cells, and marginally reduced in shVPS35b cells (Fig. 4A). Of note, VPS35^KO^ cells showed a marked decrease in mRNA expression of all MCM2-7 genes (Fig. 4B), which was consistent with the protein expression in VPS35^KO^ cells (Fig. 3C and D). These results suggested that VPS35 was involved in the mRNA expression of MCM2-7.

### Re-expression of VPS35 in VPS35^KO^ cells rescues the expression of MCM2-7

**Figure 5:**
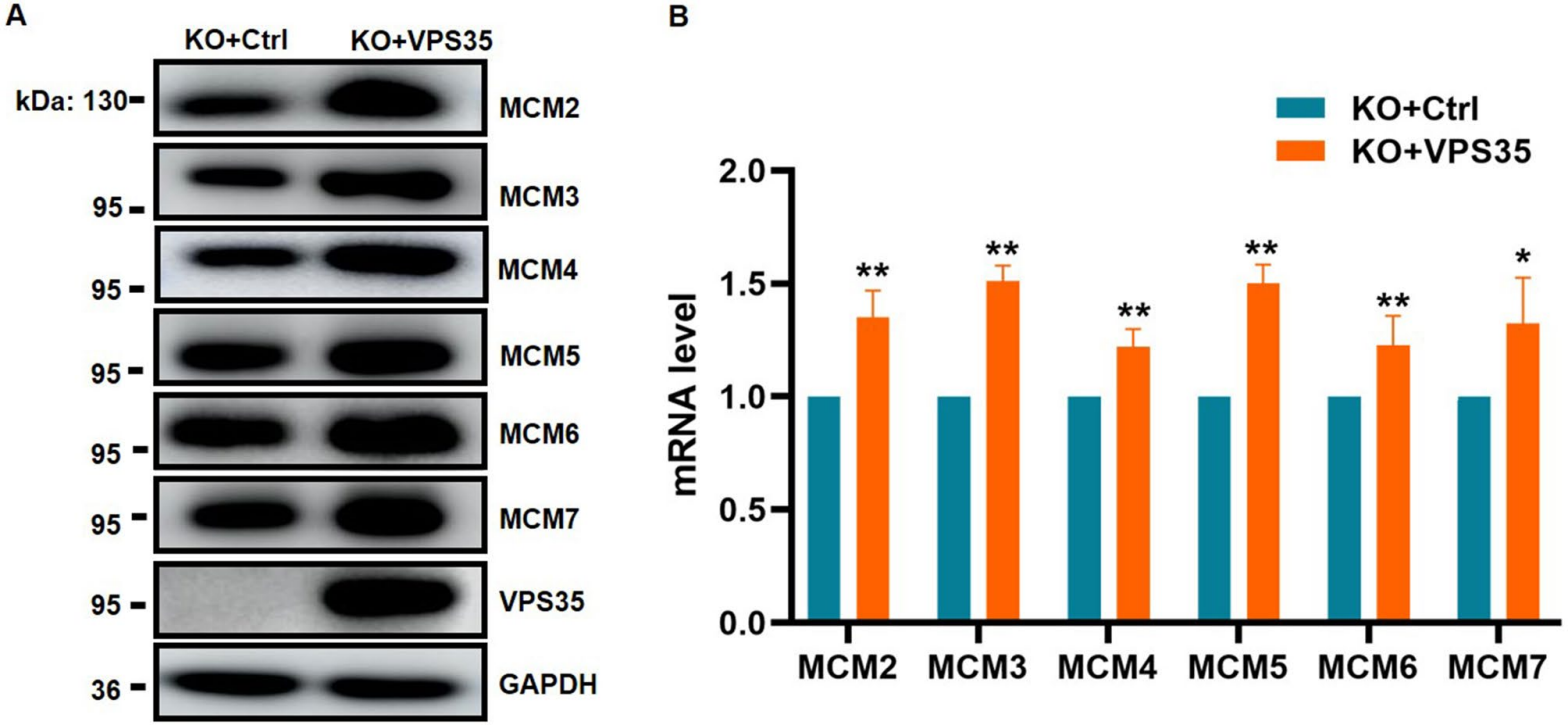
VPS35 rescued VPS35^KO^ cells restore the expression of MCM2-7 genes. VPS35^KO^ HeLa cells were infected with pLenti6.3-Flag.VPS35 or pLenti6.3-Flag.vector control lentivirus and selected with blasticidin. The protein (**A**) and mRNA (**B**) levels of MCM genes were examined in these stable cell lines by western blotting and qRT–PCR analysis, respectively. *, *p* < 0.05 vs. KO + Ctrl; **, *p* < 0.01 vs. KO + Ctrl.

To avoid the phenotypic association with off-target effects, we overexpressed wild-type VPS35 in VPS35^KO^ HeLa cells using a lentivirus mediated stable overexpression system. Importantly, VPS35 rescued HeLa cells showed a dramatic increase in mRNA and protein levels of MCM2-7 in contrast to VPS35^KO^ control cells (Fig. 5A and B). Taken together, these data provided strong evidence supporting the ability of VPS35 to promote the expression of MCM2-7 genes.

### VPS35 promotes cell proliferation

**Figure 6:**
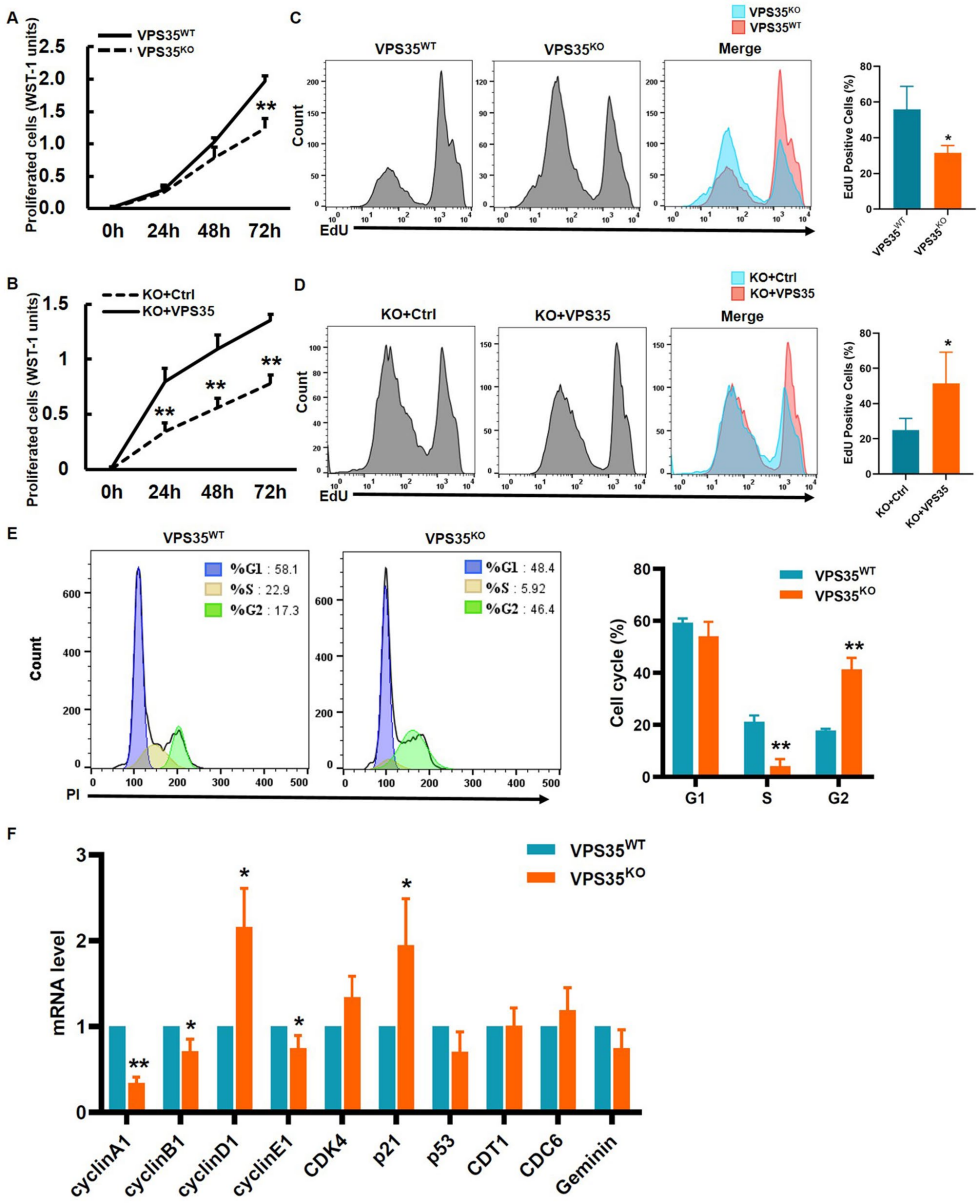
VPS35 enhances the proliferation potential of HeLa cells. (**A**–**D**) HeLa cell proliferation was measured by the WST-1 assay (**A** and **B**) and EdU incorporation assay (**C** and **D**). The ratio of EdU-positive cells to total cells was quantified by FlowJo software from three independent experiments, and expressed as the mean ± S.D. (right panels of **C** and **D**).*, *p* < 0.05; **, *p* < 0.01. (**E**) DNA content was measured by FACS analysis using propidium iodide (PI) staining. The cell cycle distribution was analyzed using FlowJo software. Histograms show the percentage of the cell cycle in HeLa cells. **, *p* < 0.01 vs. VPS35^WT^. (**F**) The mRNA levels of cell cycle-related factors, including cyclin A1, cyclin B1, cyclin D1, cyclin E1, CDK4, p21, p53, CDC6, CDT1 and Geminin, were examined by qRT–PCR. Each gene expression value is expressed as the mean ± S.D. derived from three independent experiments. *, *p* < 0.05 vs. VPS35^WT^; **, *p* < 0.01 vs. VPS35^WT^.

Considering the association of MCM2-7 gene expression and cell proliferation, we next examined whether VPS35 could affect cell proliferation. A subsequent cell viability assay showed that knockout of VPS35 significantly repressed the proliferation of HeLa cells (Fig. 6A), and overexpression of VPS35 enhanced cell proliferation in VPS35^KO^ cells (Fig. 6B). In agreement with the WST-1-cell proliferation assay, VPS35^KO^ cells displayed a marked decrease in the number of EdU-positive cells compared with VPS35^WT^ cells (Fig. 6C), indicating that the loss of VPS35 resulted in a reduction in S-phase entry. Moreover, re-expression of VPS35 led to a marked increase in the number of EdU-positive VPS35^KO^ cells (Fig. 6D), suggesting that VPS35 was required to maintain cell proliferation rates. Consistently, flow cytometry cell cycle analysis showed a dramatically decreased percentage of VPS35^KO^ cells in S-phase and a markedly increased percentage in G2-phase (Fig. 6E), suggesting that cell cycle arrest occurred following VPS35 knockout. Following further examination of cell cycle-related factors, VPS35 knockout reduced mRNA levels of cyclin A1, cyclin B1 and cyclin E1 but increased those of cyclin D1 and p21 (Fig. 6F). There were no changes in the mRNA levels of CDK4, CDC6, CDT1, Geminin or p53 (Fig. 6F). Upregulation of negative regulator p21 and downregulation of positive regulators, such as cyclin A1, cyclin B1 and cyclin E1, demonstrated that VPS35 knockout indeed triggered cell cycle arrest. Taken together, these data suggested that VPS35 contributed to cell proliferation.

### VPS35 contributes to the maintenance of genome stability

**Figure 7:**
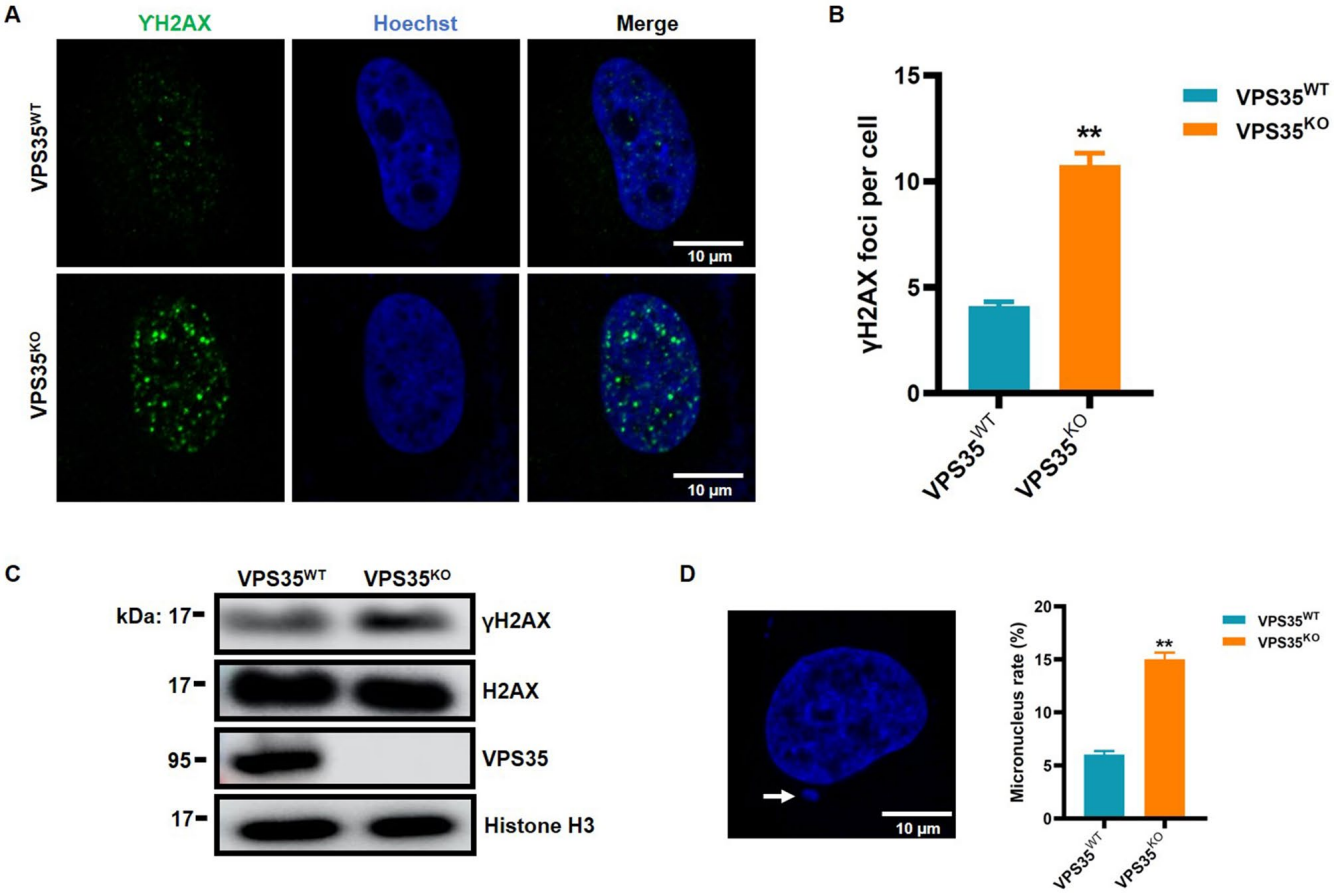
VPS35 contributes to the maintenance of genome stability. (**A**) Representative confocal images of HeLa cells labeled with anti-γH2AX antibody. (**B**) Numbers of γH2AX foci per cell were calculated for at least 200 cells of each group and presented as the mean ± S.D. **, *p* < 0.01 vs. VPS35^WT^. (**C**) The nuclear protein extracts were isolated, and the level of γH2AX was examined by western blotting. (**D**) A micronucleus assay was performed to assess genomic instability. At least 500 cells were examined, and the frequency of cells with micronuclei was calculated. **, *p* < 0.01 vs. VPS35^WT^.

Due to the essential role of MCM2-7 proteins in the maintenance of genome stability^34,35^, we next detected the effect of VPS35 on genomic aberrations. First, the number of foci of γH2AX (phosphorylation of the histone variant H2AX at serine 139), which serves as a biomarker of DNA damage, was assessed. VPS35^KO^ cells displayed an increased number of γH2AX foci under unchallenged conditions when compared with VPS35^WT^ cells (Fig. 7A and B), suggesting that VPS35 knockout resulted in spontaneous DNA damage and genomic instability. Consistent with this finding, the level of γH2AX in VPS35^KO^ cells was increased compared with that in VPS35^WT^ cells (Fig. 7C). Additionally, an increased number of micronuclei, an indicator of genomic instability, was observed in VPS35^KO^ compared with VPS35^WT^ cells (Fig. 7D). Collectively, these results demonstrated that VPS35 was required for the maintenance of genome stability.

## Discussion

In this study, iTRAQ-based quantitative proteomics identified the downregulation of several members of the MCM2-7 complex in VPS35-depleted cells. Moreover, we validated that the loss of VPS35 reduced the mRNA and protein expression of MCM2-7 genes in HeLa cells. To our knowledge, this is the first direct demonstration that VPS35 participates in the regulation of MCM2-7 gene expression. Further investigations in other cell lines are needed to confirm our results.

Of note, our previous findings indicated that VPS35 is involved in regulating the nuclear localization and accumulation of FAM21, a direct interacting partner of VPS35, but does not affect its protein level^21^. The mechanism underlying VPS35-mediated FAM21 nuclear accumulation remains to be elucidated. Nevertheless, we found that loss of VPS35 markedly diminished the mRNA and protein levels of MCM2-7 genes, in contrast to the role of VPS35 in the regulation of FAM21 nuclear localization. The expression of all members of the endogenous MCM2-7 genes is regulated by the transcription factor E2F^36^, and the ERK/MAPK signal transduction pathway is essential for E2F-dependent MCM expression^37^. Interestingly, a previous study has shown that VPS35 promotes the activation of downstream ERK/MAPK signaling through the regulation of N-Ras trafficking^38^. Therefore, VPS35 could regulate MCM2-7 gene expression through activation of ERK/MAPK pathway by trafficking of N-Ras. It is worth noting that VPS35 has been reported to directly participate in the activation of the transcription factor STAT3^39^. VPS35 interacts with TRIM27 and STAT3, and regulates TRIM27-mediated STAT3 activation. Moreover, VPS35 knockdown diminishes IL-6-induced activation of STAT3 target genes, including those encoding SOCS3 and FOS. In addition, nuclear FAM21 has been observed to modulate gene transcription through association with the transcription factor NF-κB^21^. Thus, it remains possible that VPS35 could be involved in the transcriptional regulation of MCM2-7 directly or indirectly through transcription factor activation. Further investigations are necessary to address the molecular mechanisms underlying the function of VPS35 in MCM2-7 gene expression.

Recent studies have shown that VPS35 is associated with cell proliferation and plays an oncogenic role in tumorigenesis through retromer-dependent endosomal trafficking^13,38^. Consistently, we found that VPS35 knockout significantly repressed cell proliferation and induced cell cycle arrest in HeLa cells. Notably, we identified and validated the downregulation of MCM2-7 proteins in VPS35-deficient HeLa cells. The MCM2-7 complex plays crucial roles as a DNA helicase and organizing center in DNA replication initiation and elongation, and its expression levels are correlated with the cell proliferation state^40^. MCM proteins are significantly downregulated when the cells exit the cell cycle into quiescence, and are upregulated in proliferating cells as well as in cells with the potential to proliferate^40^. Overexpression of MCM proteins has been detected in various cancer tissues and carcinoma cells^33^, and knockdown of MCM genes significantly inhibits tumor cell proliferation^41–44^. In addition to their essential roles in DNA replication, MCM proteins are involved in cell cycle progression to promote cell proliferation. MCM2 knockdown reduces cyclin D1 and CDK4 expression, increases p21 and p53 expression, and triggers G1/S arrest^41^. Knockdown of MCM3 inhibits cyclin A1 expression and causes G1 arrest^42^. Silencing of MCM6 leads to a delay in S/G2-phase progression with downregulation of Cyclin A, Cyclin B1, Cyclin D1, Cyclin E, CDK2 and CDK4^43^. Moreover, MCM7 knockdown reduces cyclin D1, cyclin E2 and CDK2 and inhibits cell proliferation^44^. Our findings indicated that VPS35 was involved in regulating the expression of cell cycle-related factors such as cyclin A1, cyclin B1, cyclin E1 and p21, suggesting that VPS35 might regulate these cell cycle regulators through MCM2-7 genes. Taken together, this study provides potential mechanistic insight into the roles of VPS35 in tumor cell proliferation.

Accurate DNA replication and rapid DNA damage repair are essential for the maintenance of genome integrity. The deficiency of MCM2-7 genes induces chromosome instability in mouse cells^34,35^. Of note, a previous study reported that the MCM2-7 complex promotes DNA double-strand break (DSB) repair through both homologous recombination (HR) and nonhomologous end-joining (NHEJ) pathways^45^. Moreover, the DNA replication licensing factor CDT1, which is required for the loading of MCM2-7 proteins onto DNA, is recruited to DSB sites and involved in the DNA damage response^46^. Our findings show that VPS35 knockout causes spontaneous DNA damage and genomic instability, suggesting that VPS35 may promote the expression of MCM2-7 genes to maintain genomic stability.

In summary, the finding that VPS35 participates in the regulation of MCM2-7 genes reveals a link between the biological roles of VPS35 and cell proliferation. However, the molecular mechanism underlying the effects of VPS35 on MCM2-7 gene expression remains to be determined. Given the presence of VPS35 in the cell nucleus, further investigations are necessary to clarify whether nuclear VPS35 indeed regulates the expression of MCM genes and to elucidate the detailed mechanisms. Therefore, the present study paves the way for further investigations on the functional significance of VPS35 or retromer proteins in the nucleus.

## Methods

### Cell culture

HeLa and HEK293T cells were purchased from the Cell Bank of Chinese Academy of Sciences (Shanghai, China). Cells were cultured in Dulbecco’s modified Eagle media (DMEM), containing 10% fetal bovine serum (FBS) in a humidified incubator with 5% CO_2_ at 37 °C.

### Generation of VPS35 knockdown stable cell lines

Lentivirus-mediated short hairpin RNA (shRNA) for stable VPS35 knockdown in HeLa cells has been described^21^. Briefly, two pairs of complementary shRNA oligonucleotides targeting the human VPS35 coding sequence were cloned into the pLKO.1 lentiviral vector. HEK293T cells were cotransfected with pLKO.1-shVPS35a, shVPS35b or scrambled control with psPAX2 (Addgene) and pMD2.G (Addgene) using polyethyleneimine (PEI, Polysciences, 23966). HeLa cells were infected with collected lentiviral particles and selected using 2 μg/mL puromycin (Thermo Fisher, A1113803) for 2 weeks. Pooled resistant clones were used as stable HeLa cell clones after validation of successful VPS35 suppression.

### CRISPR/Cas9-mediated knockout of VPS35 and rescue of VPS35 expression

The VPS35 knockout (VPS35^KO^) HeLa cell line was generated via the CRISPR/Cas9 system. An RNA guide sequence (GTAGGACAAAAACAAGCTTA) targeting exon 3 of the human VPS35 gene was used as a target and cloned into the pSpCas9(BB)-2A-Puro (PX459) V2.0 (Addgene, 62988) vector. The sgRNA/Cas9 expression construct was transiently transfected into HeLa cells. Twenty-four hours after transfection, the cells were treated with puromycin for 48 h. Then, 100–200 cells were plated onto 10-cm dishes to acquire single-cell clones. After single-cell expansion, at least 30 cell clones were selected, and western blotting was used to verify VPS35 knockout. For stable reconstituted expression of VPS35, we cloned its full-length coding region with an N-terminal Flag-tag in the pLenti6.3 vector. The resulting pLenti6.3-Flag.VPS35 plasmid and pLenti6.3-Flag vector were packaged into lentiviral particles using HEK293T cells, and then these lentiviruses were used for infection of VPS35^KO^ HeLa cells. The infected cells were selected in culture media supplemented with 20 μg/mL blasticidin (Thermo Fisher, A1113903) for 2 weeks.

### Nuclear protein extraction

The nuclear protein extraction protocol was adapted from Dignam JD et al.^47^. Briefly, cells were harvested by centrifugation at 2000 rpm for 5 min, and the supernatant was discarded. The cell pellet was suspended in Buffer A (10 mM HEPES, 1.5 mM MgCl_2_, 10 mM KCl and 0.5 mM DTT) with protease inhibitors (1 mM phenylmethylsulfonyl fluoride, 10 µg/mL aprotinin, 10 µg/mL leupeptin and 1 mM Na_3_VO_4_), and collected by centrifugation. The cells were resuspended in Buffer B (10 mM HEPES, 1.5 mM MgCl_2_, 150 mM KCl, 0.1% NP-40 and 0.5 mM DTT) with protease inhibitors, and allowed to stand on ice for 10 min. The samples were centrifuged at 6500 rpm for 3 min at 4 °C, and the supernatant, containing cytoplasmic proteins, was removed or collected. The nuclear pellet was washed twice with Buffer A containing protease inhibitors, and the pellet was collected by centrifugation. The resulting cell pellet was resuspended in Buffer C (10 mM HEPES, 25% glycerol, 420 mM NaCl, 1.5 mM MgCl_2_, 0.2 mM EDTA and 0.5 mM DTT) with protease inhibitors and mixed on a rotating wheel at 4 °C for 30 min. The samples were centrifuged at 12,000 × *g* for 10 min at 4 °C, and the supernatant containing nuclear proteins was collected. For iTRAQ labeling, the nuclear protein extraction from shVPS35a and shControl stable HeLa cells was assessed as two biological replicates.

### iTRAQ labeling and NanoLC–MS/MS analysis

The protein concentration was determined with a Bio–Rad protein assay kit based on the Bradford method using BSA as a standard. One hundred micrograms of protein from each sample was desalted by ultrafiltration with triethylammonium bicarbonate buffer, digested with 3.3 μg of trypsin per 100 μg of protein at 37 °C for 24 h, and then labeled according to the instructions supplied with the iTRAQ Reagents-8PLEX Multiplex Kit (SCIEX). Then, the two shControl samples were labeled with 113 and 116 iTRAQ tags, while two shVPS35a samples were labeled with 114 and 119 iTRAQ tags, respectively.

Next, the iTRAQ-labeled samples were pooled and fractionated by strong cation exchange chromatography on a Luna SCX column (250 × 4.60 mm, 5 μm, 100 Å, Phenomenex) with a linear gradient of 0–2 M KCl (10 mM KH_2_PO_4_, pH 3.0; 25% acetonitrile) over 81 min at a flow rate of 1 mL/min. According to the chromatography results, the collected fractions were recombined into 16 fractions and then freeze-dried. Subsequently, each of the freeze-dried fractions from the SCX column was redissolved in 0.1% formic acid aqueous solution and then desalted on strata-X C18 (Phenomenex).

The desalted peptide mixture was loaded onto an Acclaim PePmap C18 reversed-phase column (75 μm × 2 cm, 3 μm, 100 Å Thermo Fisher Scientific) and separated with reversed-phase C18 column (75 μm × 10 cm, 5 μm, 300 Å, Agela Technologies) mounted on a Dionex ultimate 3000 NanoLC system. Peptides were eluted using a gradient of 5–80% (v/v) acetonitrile in 0.1% formic acid over 45 min at a flow rate of 300 nL/min combined with a Q Exactive mass spectrometer (Thermo Fisher Scientific). The eluates were directly entered into Q Exactive MS (Thermo Fisher Scientific) with settings in positive ion mode and data-dependence with a full MS scan from 350 to 2000 m/z, full scan resolution of 70,000, MS/MS scan resolution for higher collision energy dissociation (HCD) spectra of 17,500, MS/MS scan with a minimum signal threshold of 1E + 5, and isolation width of 2 Da. For HCD, the normalized collision energy was set to 28.

### Proteomic and bioinformatics analysis

Acquired raw data were searched using Mascot (version 2.3.0; Matrix Science) embedded into Proteome Discoverer software (version 1.4; Thermo Scientific) against the UniProt-Human_9606 Database. The parameters used were as follows: peptide tolerance = 15 ppm; MS/MS tolerance = 20 mmu; enzyme = trypsin; max missed cleavage = 1; minimum of peptides = 1; minimum of unique peptide = 1; oxidation (M), iTRAQ 8plex (Y) as the variable modifications; and carbamidomethyl (C), iTRAQ8plex (N-term), iTRAQ 8plex (K) as the fixed modification. A decoy database search was used to calculate the false discovery rate (FDR) for peptide identification, using a screening criterion of FDR ≤ 1%. Differential protein expression, defined as an iTRAQ ratio between VPS35-depleted and control cells, was set to > 1.2 or < 0.83 and a *p* value < 0.05.

### Gene ontology (GO) analysis

The differentially expressed proteins in VPS35-depleted HeLa cells were classified according to the Gene Ontology (GO) category, including "biological process" and "cellular component", by using the gene ontology resource (http://geneontology.org/). The differentially expressed protein list selected by UniProt was input into the PANTHER classification system and thought to be significantly enriched if the *p* value was less than 0.05 using Fisher's exact test.

### Western blot analysis

Cell lysate preparation and western blot analysis were performed as previously described^48^.The primary antibodies used were as follows: anti-VPS35 (Abcam, ab226180), anti-MCM2 (Bethyl Laboratories, A300-191A), anti-MCM3 (Santa Cruz, sc-390480), anti-MCM4 (AssayGenie, CAB13513), anti-MCM5 (Proteintech Group, 11703-1-AP), anti-MCM6 (Thermo Fisher, PA5-35922), anti-MCM7 (Santa Cruz, sc-65469), anti-H2AX (Cell Signaling Technology, 7631), anti-phospho-Histone H2A.X (Ser139) (Millipore, 05-636), GAPDH (Proteintech Group, 60004-1) and Histone H3 (Santa Cruz, sc-10809). Western blotting membranes were cut prior to probing with the indicated antibodies, and the uncropped images of western blots were shown in the Supplementary Figures. The protein signals were detected using an Amersham Imager 680 (Cytiva, USA).

### RNA extraction and qRT–PCR

RNA was extracted using the RNeasy Mini Kit (Genemark, TR01), and reverse transcription was processed with the OneScript cDNA Synthesis Kit (Abm, G233). qRT–PCR was performed with SYBR Green Premix Ex Taq II (Takara, RR820A) using Applied Biosystems QuantStudio 3 Real-Time PCR Systems. The qRT–PCR results were calculated following the 2^-ΔΔCt^ method and normalized to GAPDH. The experiments were performed in three independent experiments and expressed as the mean ± S.D. Primer sequences are presented in Supplementary Table S2.

### Cell proliferation analysis

Cell proliferation was measured by WST-1 and EdU incorporation assays. For the WST-1 assay, 3000 cells per well were seeded in a 96-well culture plate and incubated in DMEM containing 10% FBS for the indicated periods of time. Then, WST-1 (Beyotime, C0036L) was added to each well at 1:10 dilution and incubated for 3 h. The absorbance at 450 nm was measured using a microplate reader (TECAN, Spark). For EdU labeling, 5 × 10^5^ cells per well were plated into six-well culture plates, cultured for 24 h and pulsed with 10 μM EdU (Beyotime, C0071S) for 2 h at 37 °C. The cells were fixed, permeabilized, and subjected to the Click-iT reaction using the BeyoClick EdU Cell Proliferation Kit with Alexa Fluor 488 (Beyotime, C0071S) following the manufacturer’s instructions. All samples were analyzed using a FACS Calibur flow cytometer (BD Biosciences) and FlowJo software.

### Cell cycle analysis based on DNA content

HeLa cells were seeded in 6-well culture plates and cultured for 24 h. Then, the cells were harvested by trypsinization, washed twice with cold PBS and incubated with 0.5 mL staining solution (0.1% Triton X-100, 50 μg/mL PI, 0.1% natrium citrate, pH 7.6) for 15 min at room temperature in the dark. The samples were analyzed using a FACS Calibur flow cytometer (BD Biosciences) and FlowJo software.

### Immunofluorescence

Immunofluorescence staining of γH2AX and immunofluorescence were performed as previously described^49^. For the micronucleus assay, HeLa cells were seeded into 24-well plates and cultured for 48 h. Then, the cells were fixed with 4% formaldehyde, permeabilized with 0.15% Triton X-100, and stained with Hoechst 33342. Images were obtained using an LSM-710 laser scanning confocal microscope (Carl Zeiss, Germany). At least 500 cells were examined and the frequency of cells with micronuclei was calculated as a ratio of the number of cells with micronuclei to the number of total cells examined.

### Statistical analysis

Statistical analysis of all data except the MS experiment was conducted using SPSS 25.0 software. Two-tailed Student’s t tests were used for statistical analysis between two groups, and one-way analysis of variance (ANOVA) with Dunnett's multiple comparisons test was used for multiple groups. The data are expressed as the mean of three independent experiments ± the standard deviation (S.D.), with *p* < 0.05 considered statistically significant.

## Supplementary Information

Supplementary Information 1.

Supplementary Information 2.

Supplementary Information 3.

## Data Availability

The mass spectrometry proteomics data have been deposited to the ProteomeXchange Consortium via the PRIDE^50^ partner repository with the dataset identifier PXD027166.
