# Supplementary material for: ITRAQ-based quantitative proteomic analysis reveals that VPS35 promotes the expression of MCM2-7 genes in HeLa cells
Source: Sci Rep. 2022 Jun 11;12:9700. doi: 10.1038/s41598-022-13934-3 (PMC9188599; doi:10.1038/s41598-022-13934-3)

ITRAQ-based quantitative proteomic analysis reveals that VPS35 promotes the expression of MCM2-7 genes in HeLa cells

Xian Hong, Tao Wang, Juan Du, Yu Hong, Cai-Ping Yang, Wei Xiao, Yang Li, Ming Wang, He Sun, and Zhi-Hui Deng

Supplementary Fig. 1 Original images of western blotting shown in Fig. 1A and 1B

Figure 1A

Replicate 1: Used in the manuscript.

Replicate 2:

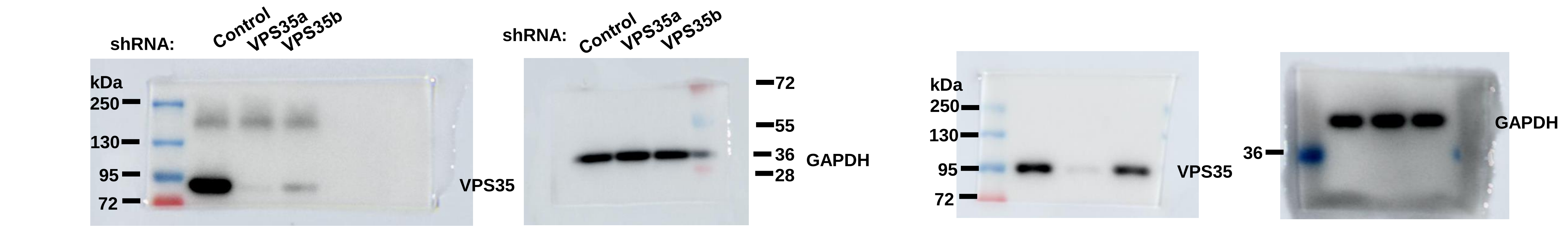

Figure 1B

Replicate 1: Used in the manuscript. Red rectangle marks the bands used.

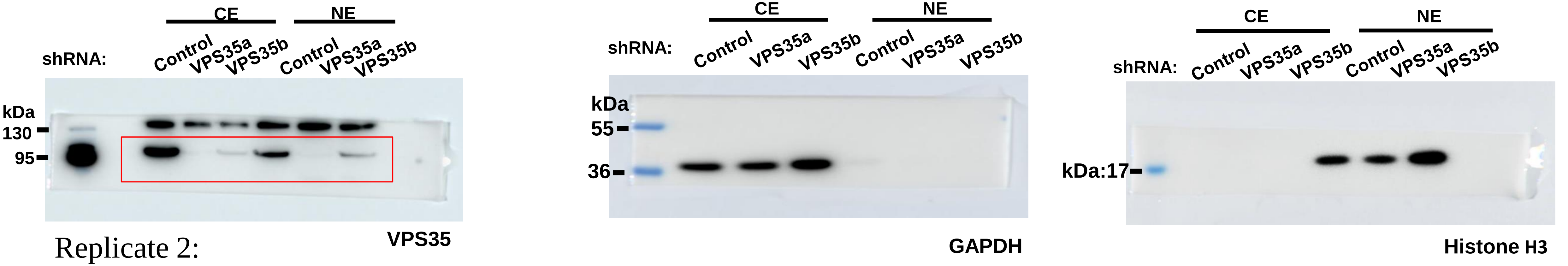

Replicate 2:

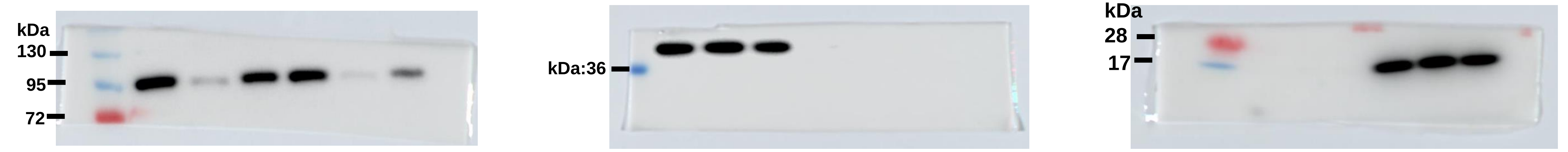

Supplementary Fig. 2 Original images of western blotting shown in Fig. 3A

Replicate 1: Used in the manuscript. Red rectangles mark the bands used.

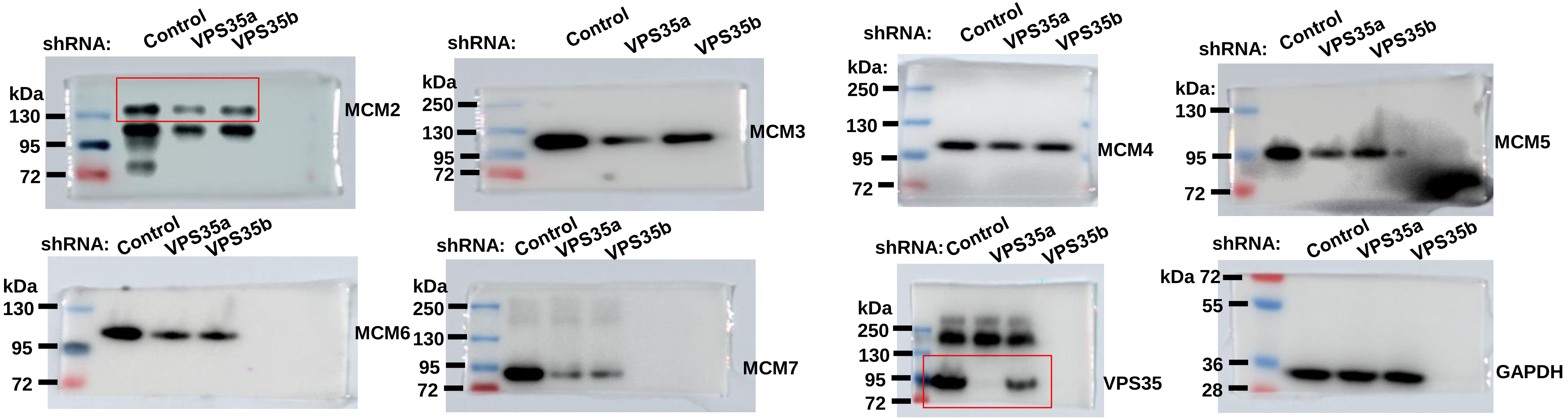

Replicate 2:

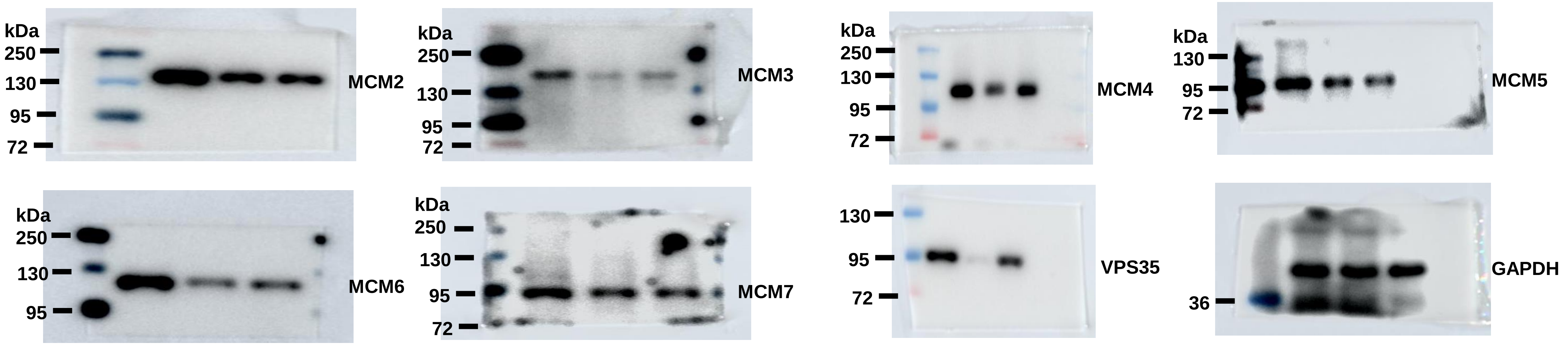

Supplementary Fig. 3 Original images of western blotting shown in Fig. 3B

Replicate 1: Used in the manuscript.

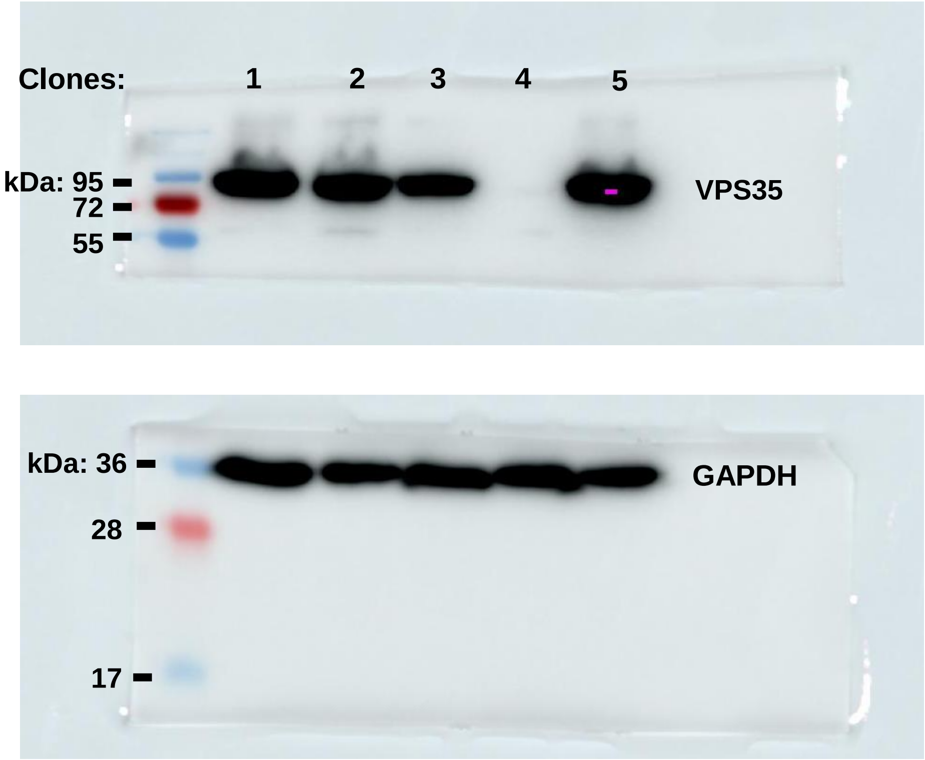

Supplementary Fig. 4 Original images of western blotting shown in Fig. 3C

Replicate 1: Used in the manuscript. Red rectangles mark the bands used.

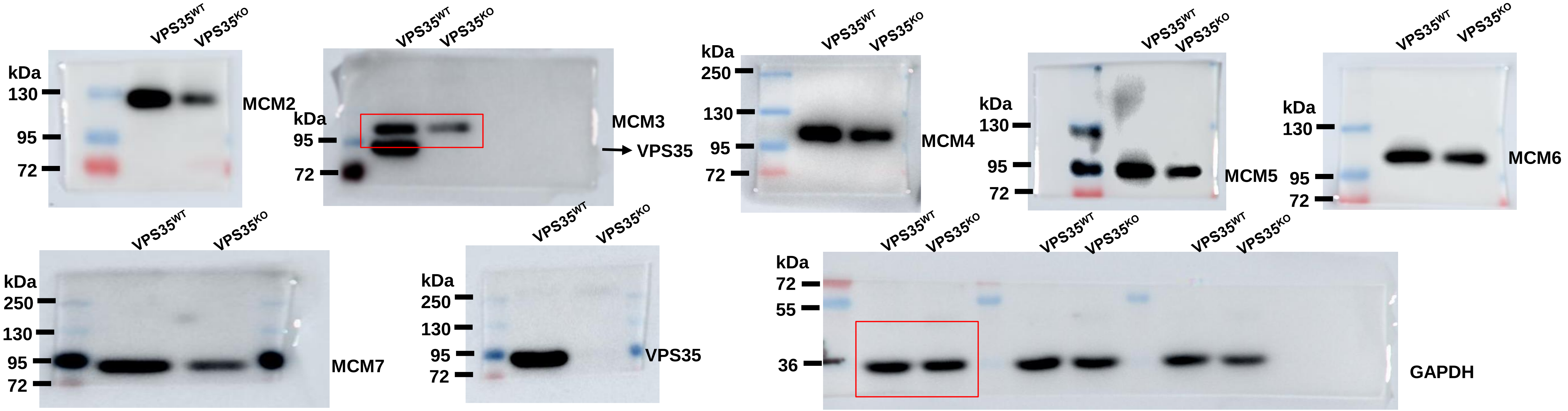

Replicate 2:

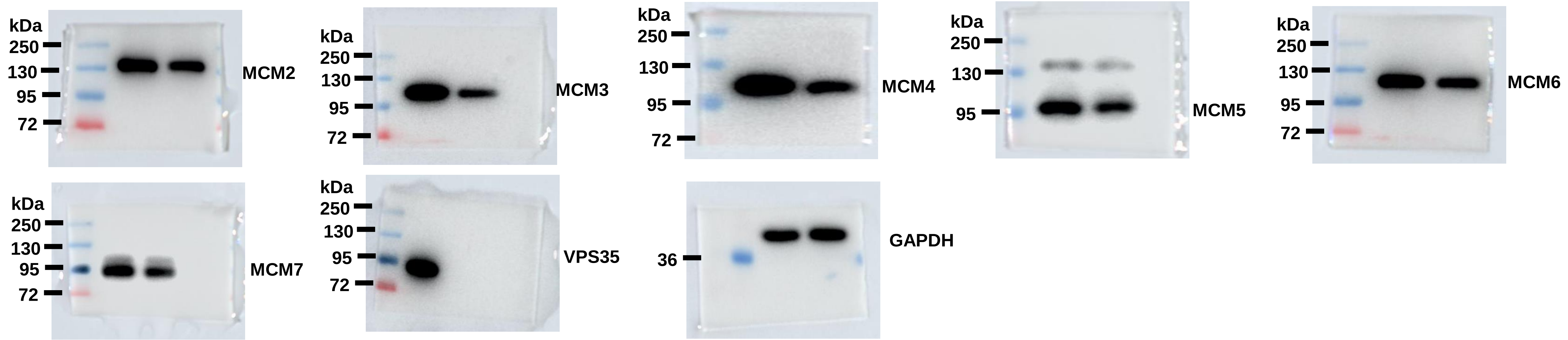

Supplementary Fig. 5 Original images of western blotting shown in Fig. 3D

Replicate 1: Used in the manuscript. Red rectangles mark the bands used.

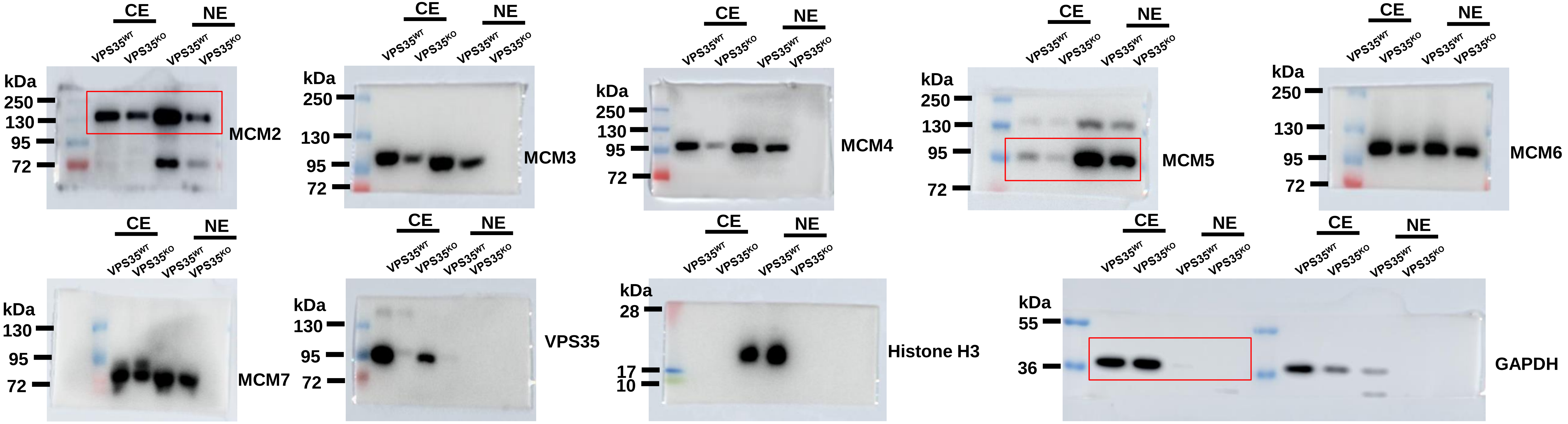

Replicate 2:

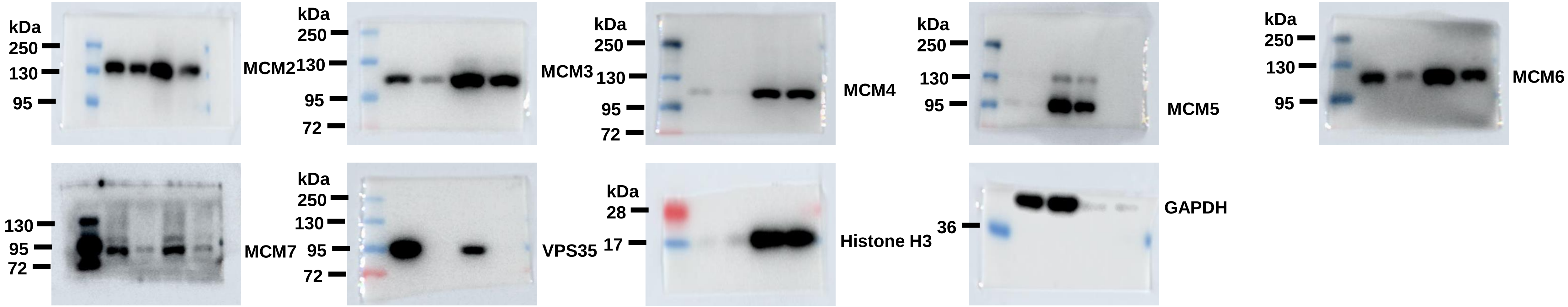

Supplementary Fig. 6 Original images of western blotting shown in Fig. 5A

Replicate 1: Used in the manuscript.

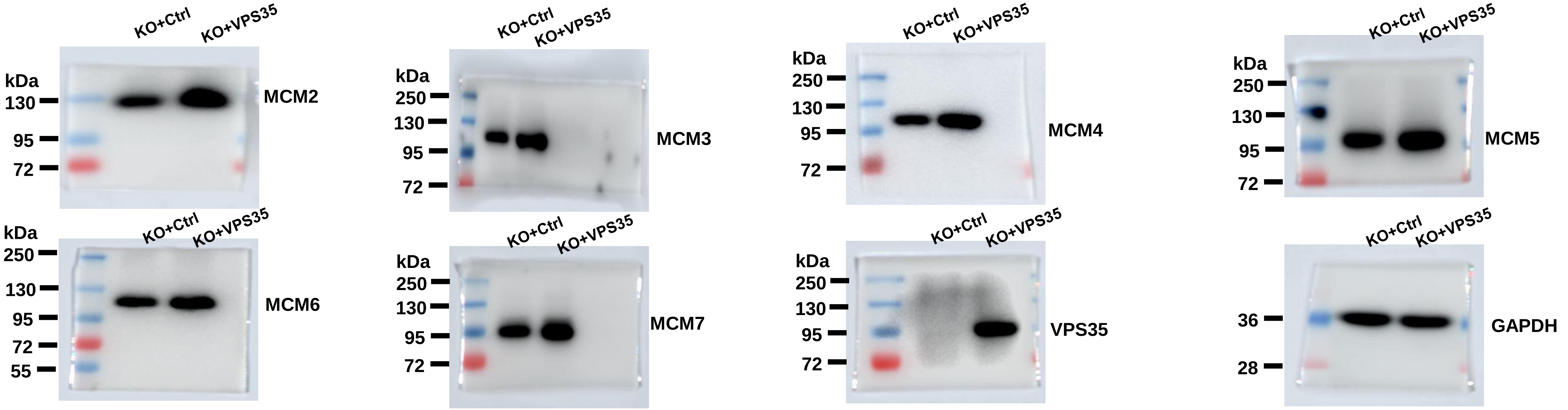

Replicate 2:

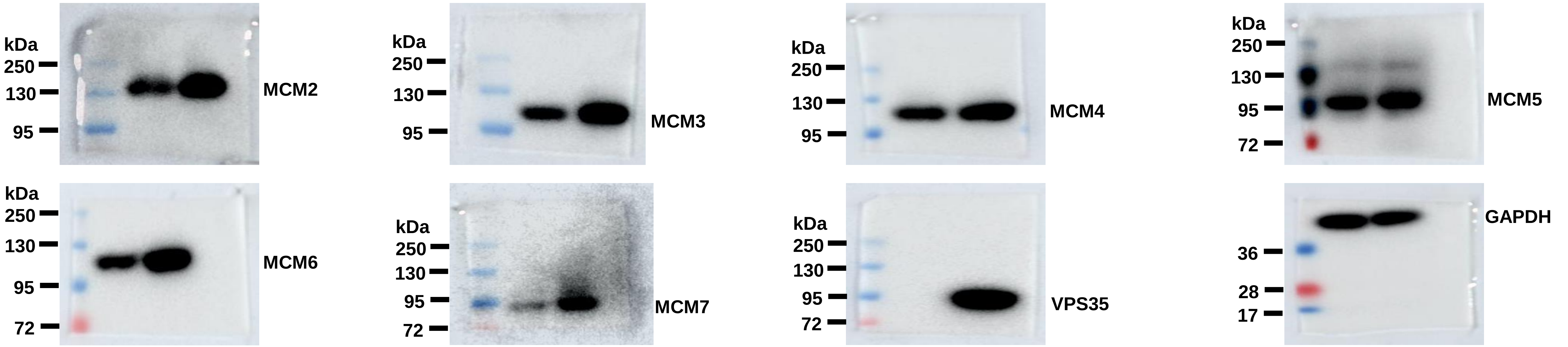

Supplementary Fig. 7 Original images of western blotting shown in Fig. 7C

Replicate 1: Used in the manuscript

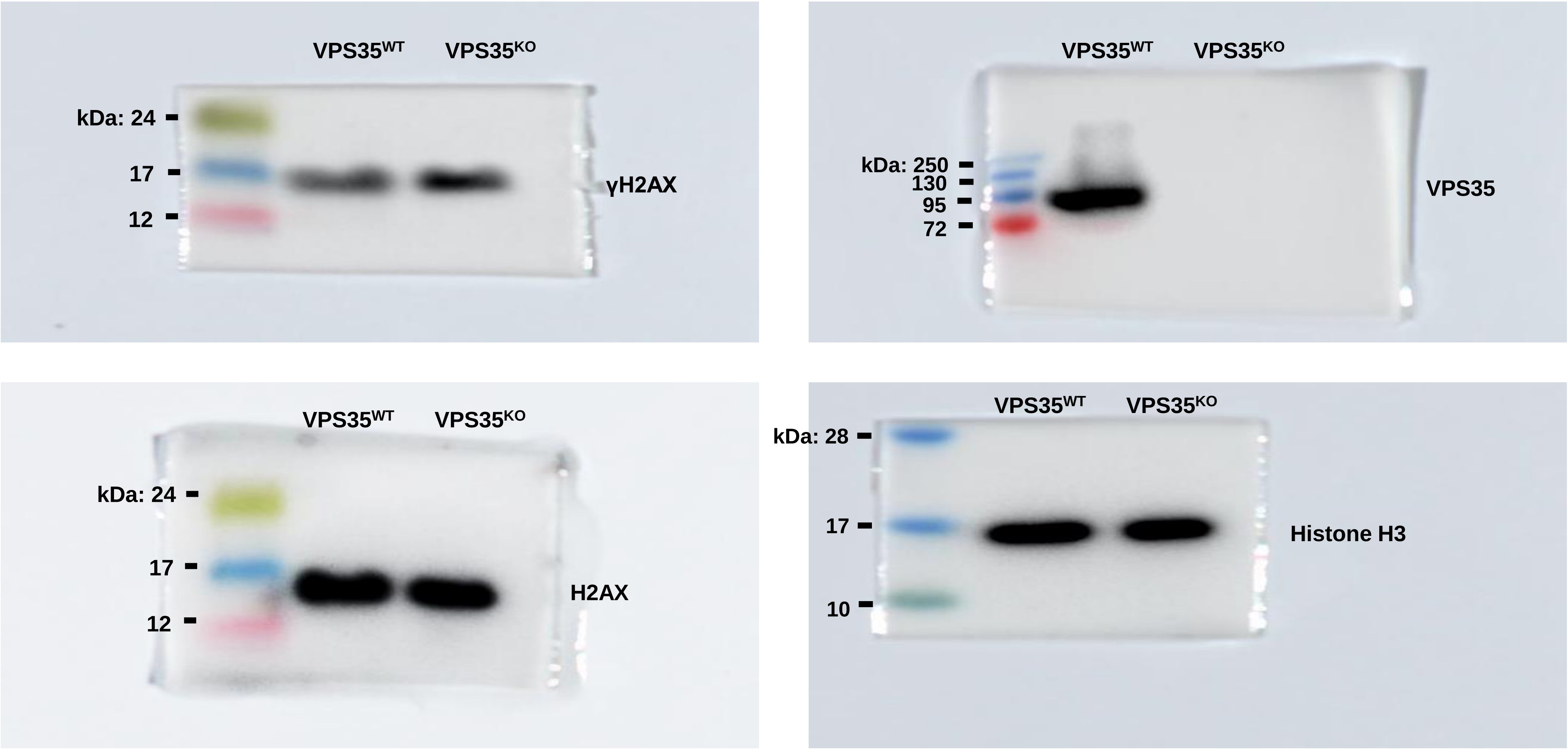

Supplement: Supplementary file 1 — Supplementary Information 1. [file 41598_2022_13934_MOESM1_ESM.pdf]
