# Supplementary material for: ITRAQ-based quantitative proteomic analysis reveals that VPS35 promotes the expression of MCM2-7 genes in HeLa cells
Source: Sci Rep. 2022 Jun 11;12:9700. doi: 10.1038/s41598-022-13934-3 (PMC9188599; doi:10.1038/s41598-022-13934-3)
Supplement: Supplementary file 3 — Supplementary Information 3. [file 41598_2022_13934_MOESM3_ESM.docx]

**Supplementary Information**

ITRAQ-based quantitative proteomic analysis reveals that VPS35 promotes the expression of MCM2-7 genes in HeLa cells

Xian Hong, Tao Wang, Juan Du, Yu Hong, Cai-Ping Yang, Wei Xiao, Yang Li, Ming Wang, He Sun, and Zhi-Hui Deng

**Supplementary Table S2. Primer sequences used for qRT-PCR**

| **Primers** | **Sequence (5’-3’)** |
| --- | --- |
| MCM2-F | ATGATCGAGAGCATCGAGAACC |
| MCM2-R | GCCAAGTCCTCATAGTTCACCA |
| MCM3 -F | GGCCTCCATTGATGCTACCTA |
| MCM3- R | ACTTTGGGACGAACTAGAGAACA |
| MCM4 -F | GACGTAGAGGCGAGGATTCC |
| MCM4 -R | GCTGGGAGTGCCGTATGTC |
| MCM5-F | GGAAGTGCAACACAGATCAGG |
| MCM5-R | AGGGACGACCTTGTCACACA |
| MCM6 -F | GAGGAACTGATTCGTCCTGAGA |
| MCM6 –R- | CAAGGCCCGACACAGGTAAG |
| MCM7 -F | CCTACCAGCCGATCCAGTCT |
| MCM7 -R | CCTCCTGAGCGGTTGGTTT |
| Cyclin A1-F  Cyclin A1-R  Cyclin B1-F  Cyclin B1-R  Cyclin D1-F  Cyclin D1-R  Cyclin E1-F  Cyclin E1-R  CDK4-F  CDK4-R  p21-F  p-21-R  p53-F  p53-R  CDC6-F  CDC6-R  CDT1-F  CDT1-R  Geminin-F  Geminin-R | TAGACACCGGCACACTCAAG  AGGAGAGATGAATCTACCAGCAT  AACTTTCGCCTGAGCCTATTTT  TTGGTCTGACTGCTTGCTCTT  GCTGCGAAGTGGAAACCATC  CCTCCTTCTGCACACATTTGAA  GCCAGCCTTGGGACAATAATG  CTTGCACGTTGAGTTTGGGT  ATGGCTACCTCTCGATATGAGC  CATTGGGGACTCTCACACTCT  CGATGGAACTTCGACTTTGTCA  GCACAAGGGTACAAGACAGTG  CAGCACATGACGGAGGTTGT  TCATCCAAATACTCCACACGC  ACCTATGCAACACTCCCCATT  TGGCTAGTTCTCTTTTGCTAGGA  GACATGATGCGTAGGCGTTTT  GAGCTGGTAATCTGACCTCCT  GCCCTGGGGTTATTGTCCC  AGCGCCTTTCTCCGTTTTTCT |
| GAPDH-F | AATGAAGGGGTCATTGATGG |
| GAPDH-R | AAGGTGAAGGTCGGAGTCAA |
